# Value of urinary KIM-1 and NGAL combined with serum Cys C for predicting acute kidney injury secondary to decompensated cirrhosis

**DOI:** 10.1038/s41598-018-26226-6

**Published:** 2018-05-21

**Authors:** Lei Lei, Liang Ping Li, Zhen Zeng, Jing Xi Mu, Xue Yang, Chao Zhou, Zhi Lan Wang, Hu Zhang

**Affiliations:** 1 Department of Gastroenterology and Hepatology, Sichuan Academy of Medical Sciences & Sichuan Provincial People’s Hospital, Chengdu, China; 2 Department of Gastroenterology and Hepatology, West China Hospital, Sichuan University, Chengdu, China

## Abstract

Urinary kidney injury molecule-1 (KIM-1), neutrophil gelatinase-associated lipocalin (NGAL), and serum cystatin C (Cys C) are biomarkers of acute kidney injury (AKI). However, the efficacy of combining these indices to diagnose decompensated cirrhosis is unknown. This study involved 150 patients divided into AKI and non-AKI, and healthy individuals. Urinary KIM-1 and NGAL, serum Cys and creatine, and glomerular filtration rate (GFR) were compared based on Child-Pugh liver function class. Urinary KIM-1 and NGAL concentrations and serum Cys C levels were significantly higher in patients with AKI secondary to decompensated cirrhosis than in those with AKI not secondary to decompensated cirrhosis (*p* < 0.01). These were significantly associated with higher kidney injury index stages (*p* < 0.01) and negatively correlated with GFR in secondary AKI patients. Urinary KIM-1 and NGAL and serum Cys C increased significantly and GFR decreased as Child-Pugh class of decompensated cirrhosis significantly increased (*p* < 0.05). SCr levels were significantly increased in Child-Pugh class C patients (*p* < 0.05). Urinary KIM-1, urinary NGAL, serum Cys C, and the combined detection factor, as screening indices, could aid in the early diagnosis of AKI secondary to decompensated cirrhosis.

## Introduction

Cirrhosis is a chronic liver disease commonly identified in the clinic. During the early stages, it can occur with no clinical presentation, but patient mortality increases significantly as soon as it progresses to a decompensated stage^1^. Acute kidney injury (AKI) is one of the most severe complications of end-stage liver disease. Relevant studies^2^ have shown that the incidence of AKI is 19% among patients hospitalized due to decompensated cirrhosis, and that it is an important sign of poor prognosis and an independent predictive parameter of death. AKI primarily presents as a sharp decrease in glomerular filtration rate (GFR), rapid increases in serum creatinine (SCr) and blood urea nitrogen, and water and sodium retention. AKI secondary to decompensated cirrhosis often indicates poor prognosis, and the 30-d mortality rate is 10-fold greater than that in patients without complicating AKI^3^. Every 12-h delay in AKI diagnosis increases the in-hospital mortality rate by 2.7-fold^2^.

Currently, the mechanisms of AKI secondary to different degrees of liver injury are not clear. Overall, it is speculated that they are related to liver injury, which leads to redistribution of renal blood flow, further exacerbation of renal circulation disruption, mitochondrial damage in glomerular and tubular endothelial cells, and other factors^4^. These types of kidney injury are often functional during the early stages and can be reversed with medication if discovered and treated early; however, they can progress to hepatorenal syndrome and even to life-threatening acute or chronic kidney failure. Traditional monitoring of kidney function indices, serum creatinine, and blood urea nitrogen is extremely limited for the early diagnosis of kidney injury, and early monitoring and diagnosis of patients with AKI secondary to decompensated cirrhosis is difficult. Early monitoring of patients with this condition has always been an active research topic in this field, and thus the search for specific, sensitive early diagnostic markers is essential for its treatment and for reducing patient mortality and improving prognosis.

Investigations in recent years have revealed many new biomarkers for the diagnosis of AKI^5^. These primarily include kidney injury molecule-1 (KIM-1), cystatin C (Cys C), and neutrophil gelatinase-associated lipocalin (NGAL), among others. KIM-1 is a transmembrane protein that is not expressed in normal kidneys, but its levels increase in the urine upon kidney injury. It can reflect the degree of kidney injury, and has been used in recent years as a sensitive marker for the early diagnosis of glomerular injury^6,7^. Cys C is eliminated from the body only via the kidneys, and kidney microlesions at early stages can lead to changes in serum Cys C levels. Studies have shown that serum Cys C concentrations increase upon mild kidney injury and gradually become further elevated as the disease progresses^8,9^. NGAL is a newly identified member of the lipocalin family that is significantly expressed in injured epithelial cells. It can lead to rapid increases in NGAL secretion from glomeruli, thus increasing NGAL concentrations in the urine. In addition, its concentration often increases within 2 h of kidney injury^10,11^.

Using patients with AKI secondary to decompensated cirrhosis as the subjects, this study investigated the value of combined detection of urinary KIM-1, NGAL, and serum Cys C for the early diagnosis of AKI secondary to decompensated cirrhosis. We also evaluated the diagnostic efficacy of combined detection using these three indices, in addition to their relationship with disease progression and prognosis.

## Results

### Baseline patient characteristics

Data were collected from 150 patients with decompensated cirrhosis. The AKI group comprised 68 cases, of which 42 were males and 26 were females, and the age range was 41–79 years with an average age of 59.72 ± 10.16 years. The non-AKI group included 82 cases, of which 49 were males and 33 were females and the age range was 33–75 years with an average age of 61.47 ± 14.35 years. The healthy control group included 70 cases, of which 45 were males and 25 were females; the age range was 39–72 years with an average age of 56.87 ± 11.42 years. Liver and kidney function tests and liver ultrasound results were all within normal ranges in the healthy control group. The sex ratio, average age, and other baseline characteristics were in general agreement (all *p* > 0.05) and were comparable.

### Comparison of urinary KIM-1, urinary NGAL, serum Cys C, SCr, and GFR among the three groups

**Figure 1:**
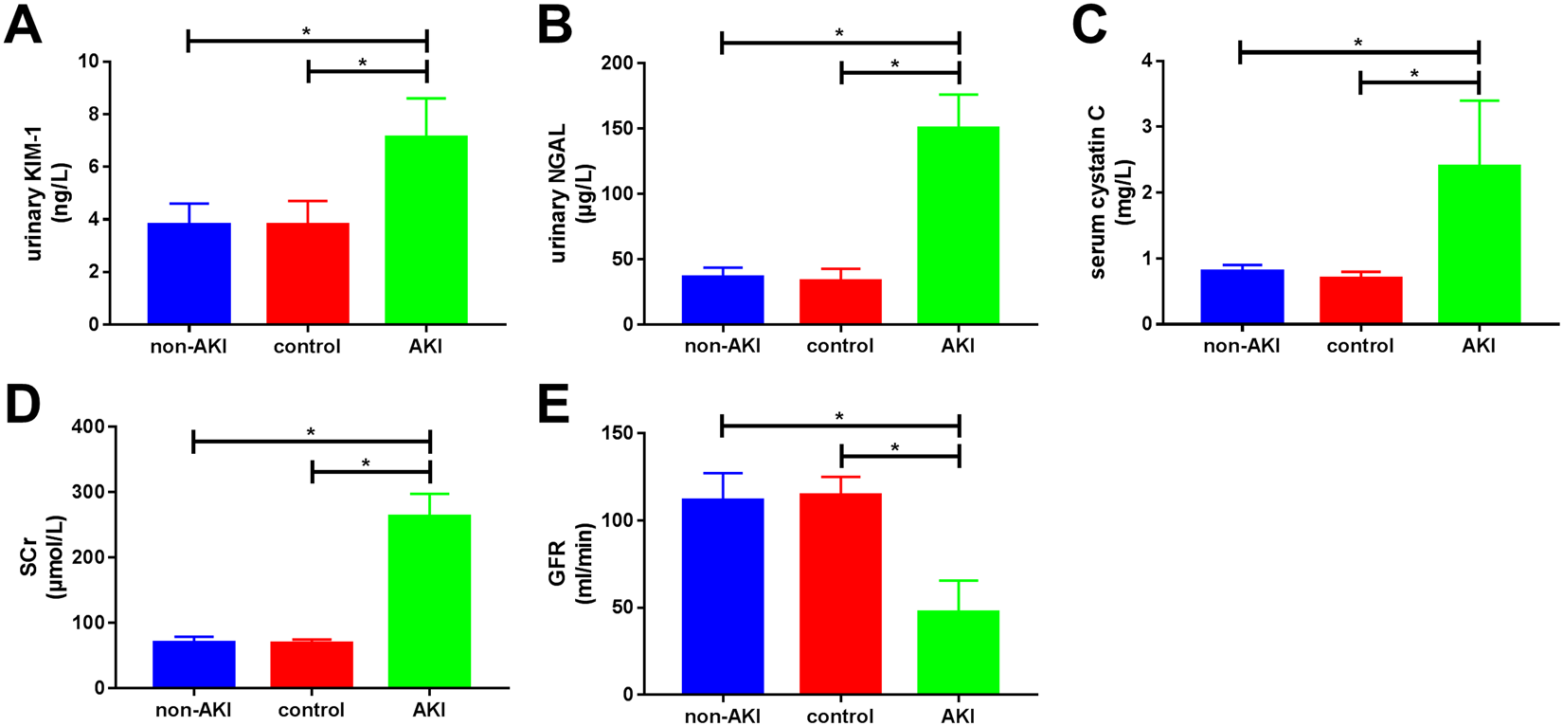
Comparison of urinary kidney injury molecule-1 (KIM-1), urinary neutrophil gelatinase-associated lipocalin (NGAL), serum cystatin C (Cys C), serum creatinine (SCr), and glomerular filtration rate (GFR) among the three patient groups. The results show that urinary KIM-1, urinary NGAL, serum Cys C, and SCr were significantly higher in the acute kidney injury (AKI) group than in the non-AKI and healthy control groups. GFR was significantly lower in the AKI group than in the non-AKI and healthy control groups. All differences were statistically significant (*p* < 0.01).

Urinary KIM-1 levels in the AKI group (7.1 ± 1.5 ng/L) were significantly higher than in those in the non-AKI group (3.5 ± 0.8 ng/L) and the control group (3.8 ± 0.9 ng/L). Urinary NGAL levels in the AKI group (150.1 ± 26.2 µg/L) were also significantly higher than those in the non-AKI group (36.2 ± 7.4 µg/L) and the control group (33.0 ± 9.7 µg/L). Similarly, serum Cys C levels in the AKI group (2.4 ± 1.0 mg/L) were significantly higher than those in the non-AKI group (0.8 ± 0.1 mg/L) and the control group (0.7 ± 0.1 mg/L). GFR was significantly lower in the AKI group (47.2 ± 18.4 ml/min) than that in the non-AKI (111.6 ± 15.6 mL/min) and control (114.6 ± 10.5 mL/min) groups. All differences were statistically significant (*p* < 0.01; Fig. 1).

### Comparison of urinary KIM-1, urinary NGAL, serum Cys C, SCr, and GFR among patients with different stages of AKI

**Figure 2:**
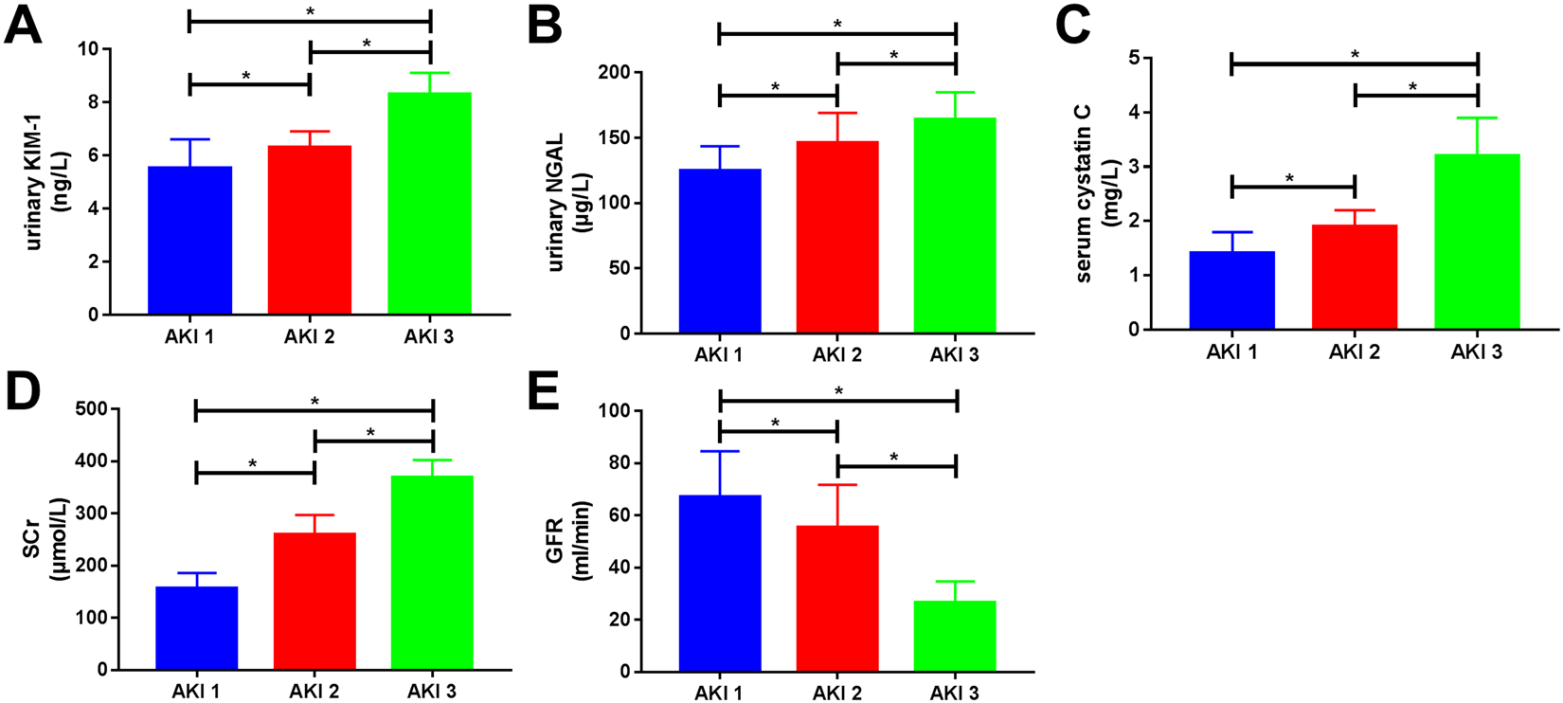
Comparison of urinary kidney injury molecule-1 (KIM-1), urinary neutrophil gelatinase-associated lipocalin (NGAL), serum cystatin C (Cys C), serum creatinine (SCr), and glomerular filtration rate (GFR) among patients in different stages of acute kidney injury (AKI). Results show that the differences in urinary KIM-1, urinary NGAL, serum Cys C, SCr, and GFR among patients in different stages were statistically significant (*p* < 0.01). As AKI stage increased, urinary KIM-1, urinary NGAL, serum Cys C, and SCr increased significantly and GFR decreased significantly.

The patients in the AKI group were divided into three groups according to the 2012 KDIGO staging criteria^12^. Fifteen patients were classified as Stage 1 AKI, 20 as Stage 2 AKI, and 33 as Stage 3 AKI. Urinary KIM-1, urinary NGAL, serum Cys C, SCr, and GFR were compared among the groups, and the results showed that as AKI clinical stage increased in severity, there were significant differences in kidney function indices at different stages. Urinary KIM-1 levels in Stage 3 AKI patients (8.3 ± 0.8 ng/L) were significantly higher than those in Stage 2 AKI patients (6.3 ± 0.6 ng/L), and urinary KIM-1 levels in Stage 2 AKI patients were significantly higher than those in Stage 1 AKI patients (5.5 ± 1.1 ng/L); all differences were statistically significant (*p* < 0.01). Urinary NGAL levels in Stage 3 AKI patients (163.8 ± 21.1 μg/L) were significantly higher than those in Stage 2 AKI patients (146.1 ± 22.9 μg/L), and urinary NGAL levels in Stage 2 AKI patients were significantly higher than those in Stage 1 AKI patients (124.7 ± 18.8 μg/L); all differences were statistically significant (*p* < 0.01). Serum Cys C levels in Stage 3 AKI patients (3.2 ± 0.7 mg/L) were significantly higher than those in Stage 2 AKI patients (1.9 ± 0.3 mg/L), and serum Cys levels in Stage 2 AKI patients were significantly higher than those in Stage 1 AKI patients (1.4 ± 0.4 mg/L); all differences were statistically significant (*p* < 0.01). Serum SCr levels in Stage 3 AKI patients (368.4 ± 34.3 μmol/L) were significantly higher than those in Stage 2 AKI patients (259.4 ± 38.0 μmol/L), and serum SCr levels in Stage 2 AKI patients were significantly higher than those in Stage 1 AKI patients (156.6 ± 29.3 μmol/L); all differences were statistically significant (*p* < 0.01). GFR in Stage 3 AKI patients (26.1 ± 8.1 mL/min) was significantly lower than in that Stage 2 AKI patients (55.4 ± 16.3 mL/min), and GFR in Stage 2 AKI patients was significantly lower than that in Stage 1 AKI patients (67.1 ± 17.4 mL/min); all differences were statistically significant (*p* < 0.01; Fig. 2).

### Analysis of correlation between urinary KIM-1, urinary NGAL, and serum Cys C and GFR among the three groups

**Figure 3:**
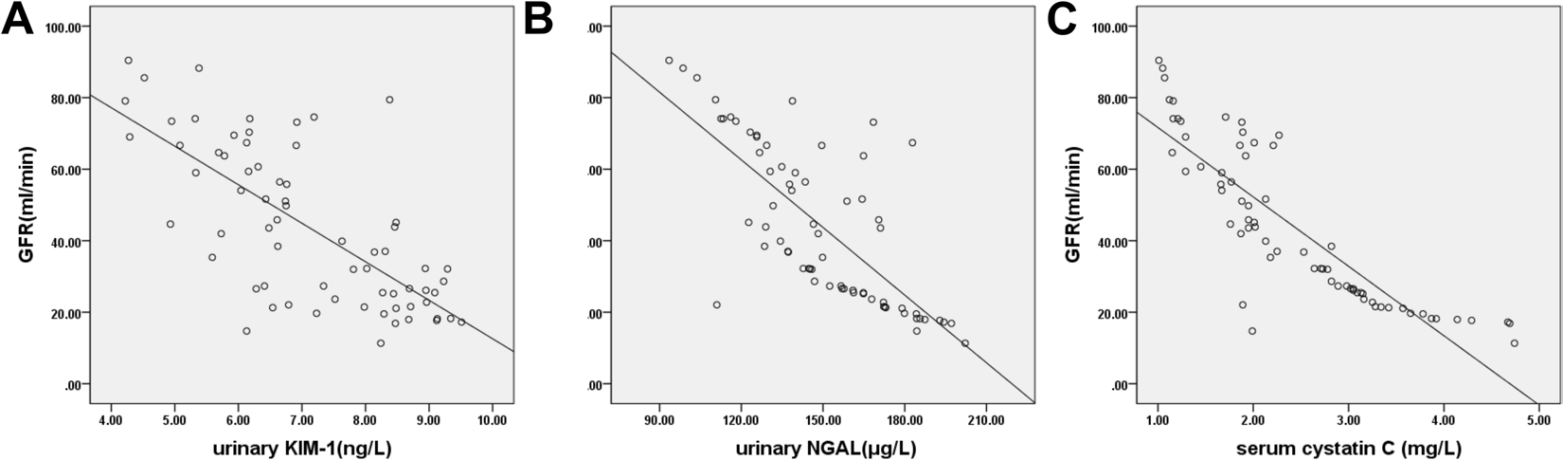
Analysis of correlation between urinary kidney injury molecule-1 (KIM-1), urinary neutrophil gelatinase-associated lipocalin (NGAL), serum cystatin C (Cys C), and glomerular filtration rate (GFR) in the acute kidney injury group (AKI) group. Urinary KIM-1, urinary NGAL, and serum Cys C were all negatively correlated with GFR in the AKI group (r-values of −0.713, −0.744, and −0.860, respectively; all *p* < 0.01).

**Figure 4:**
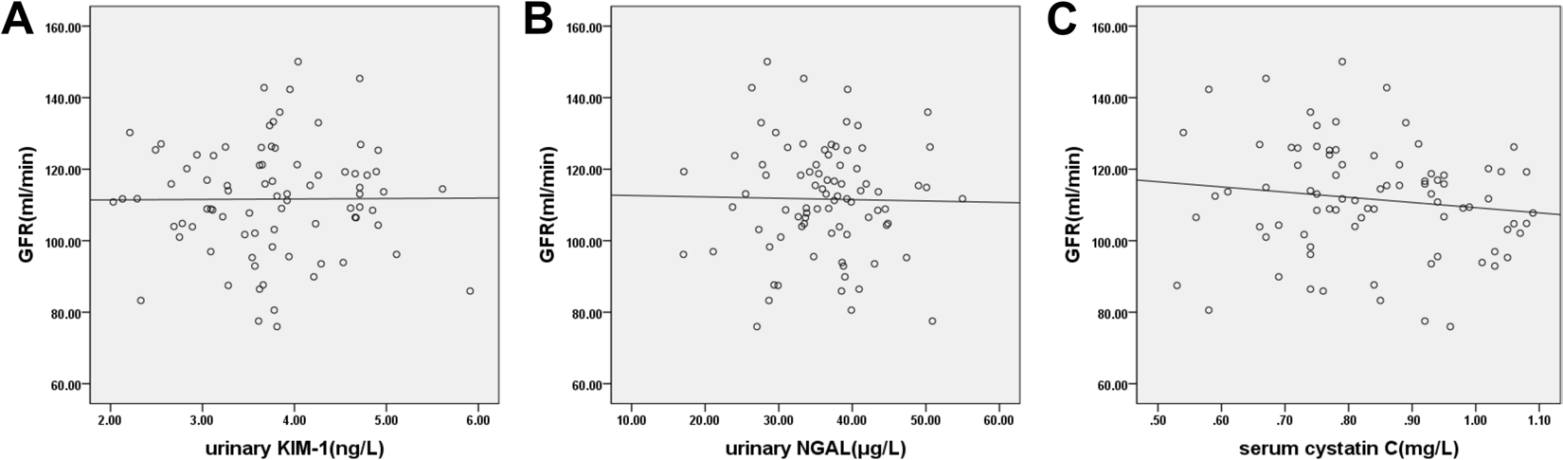
Analysis of correlation between urinary kidney injury molecule-1 (KIM-1), urinary neutrophil gelatinase-associated lipocalin (NGAL), serum cystatin C (Cys C), and glomerular filtration rate (GFR) in the non-AKI group. Urinary KIM-1, urinary NGAL, and serum Cys C were not correlated with GFR (*p* > 0.05).

**Figure 5:**
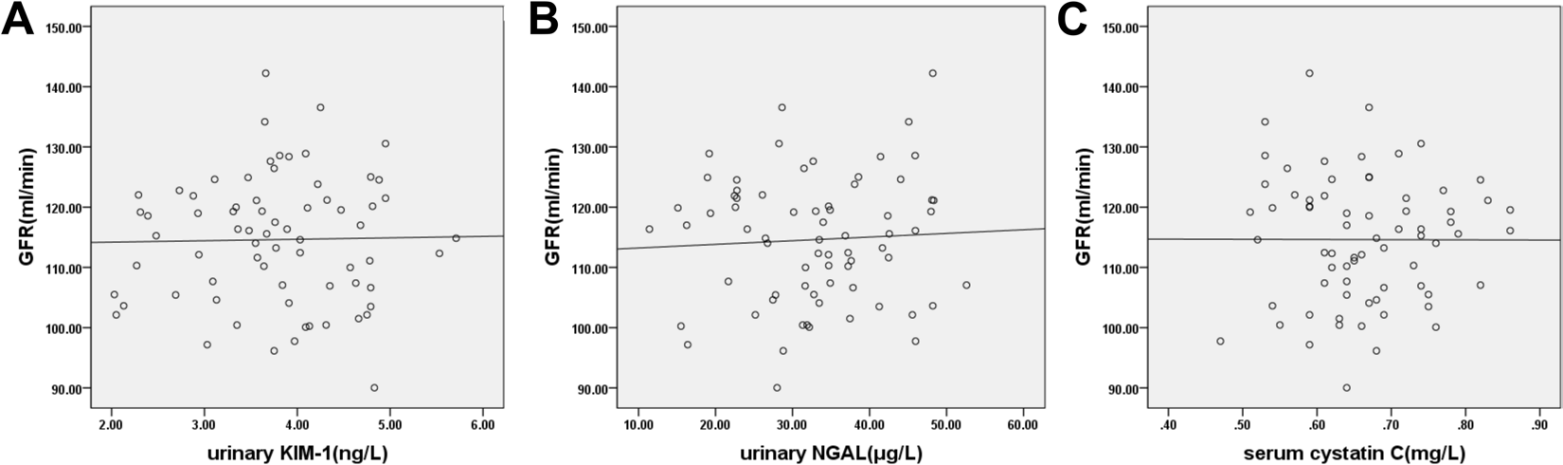
Analysis of correlation between urinary kidney injury molecule-1 (KIM-1), urinary neutrophil gelatinase-associated lipocalin (NGAL), serum cystatin C (Cys C), and glomerular filtration rate (GFR) in the healthy control group. Urinary KIM-1, urinary NGAL, and serum Cys C were not correlated with GFR (*p* > 0.05).

Pearson correlation analysis was used for the correlation analysis of bivariate normally distributed data. Analysis of the correlation between urinary KIM-1, urinary NGAL, and serum Cys C and GFR among the three groups was also performed. Results showed that in the AKI group, urinary KIM-1 levels (r = −0.713, *p* < 0.01), urinary NGAL levels (r = −0.744, *p* < 0.01), and serum Cys C levels (r = −0.860, *p* < 0.01) were negatively correlated with GFR (Fig. 3). In the non-AKI group, urinary KIM-1 levels (r = 0.007, *p* > 0.05), urinary NGAL levels (r = −0.018, *p* > 0.05), and serum Cys C levels (r = −0.135, *p* > 0.05) were not correlated with GFR (Fig. 4).In the healthy control group, urinary KIM-1 levels (r = 0.019, *p* > 0.05), urinary NGAL levels (r = 0.056, *p* > 0.05), and serum Cys C levels (r = −0.002, *p* > 0.05) were not correlated with GFR (Fig. 5).

### Comparison of urinary KIM-1, urinary NGAL, serum Cys C, SCr, and GFR in living and deceased patients in the AKI group

**Figure 6:**
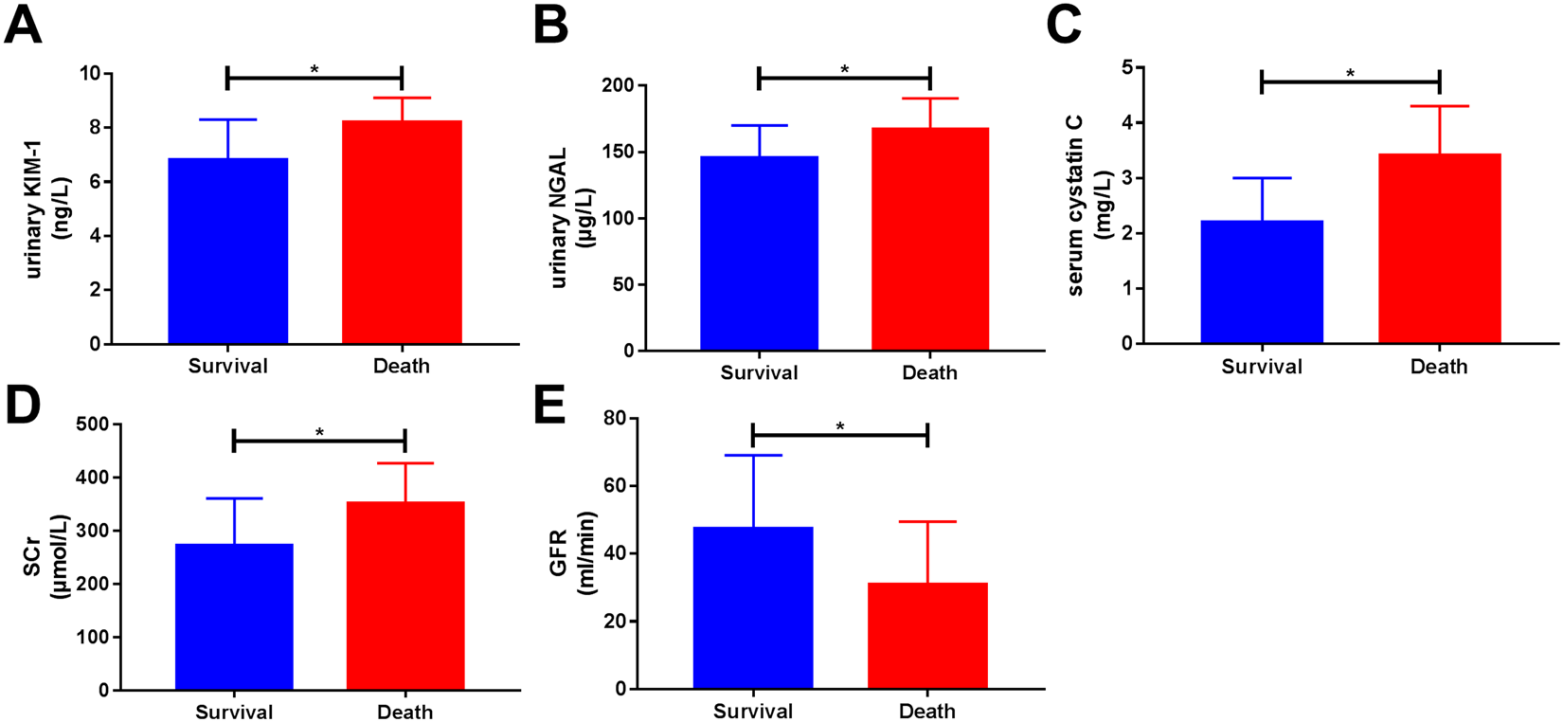
Comparison of urinary kidney injury molecule-1 (KIM-1), urinary neutrophil gelatinase-associated lipocalin (NGAL), serum cystatin C (Cys C), serum creatinine (SCr) and glomerular filtration rate (GFR) between living and deceased patients in the acute kidney injury (AKI) group. The results showed that urinary KIM-1, urinary NGAL, serum Cys C, and SCr were all significantly elevated in deceased patients compared to those in living patients (all p < 0.05), and that GFR was significantly decreased in deceased patients compared to that in living patients (*p* < 0.05).

In the AKI group, 15 of 68 patients died, resulting in a mortality rate of 22.1%. Results showed that urinary KIM-1 levels, urinary NGAL levels, serum Cys C levels, and SCr levels among the deceased patients (8.2 ± 0.9 ng/L, 166.7 ± 23.7 μg/L, 3.4 ± 0.9 mg/L, and 351.2 ± 76.1 μmol/L, respectively) were significantly higher than those among the living patients (6.8 ± 1.5 ng/L, 145.2 ± 24.8 μg/L, 2.2 ± 0.8 mg/L, and 272.2 ± 8.9 μmol/L, respectively), whereas GFR levels among the deceased patients (30.8 ± 18.7 mL/min) were significantly lower than those among the living patients (47.4 ± 21.7 mL/min). All differences were statistically significant (*p* < 0.05; Fig. 6).

### Comparison of urinary KIM-1, urinary NGAL, serum Cys C, SCr, and GFR based on Child-Pugh classes of decompensated cirrhosis

**Figure 7:**
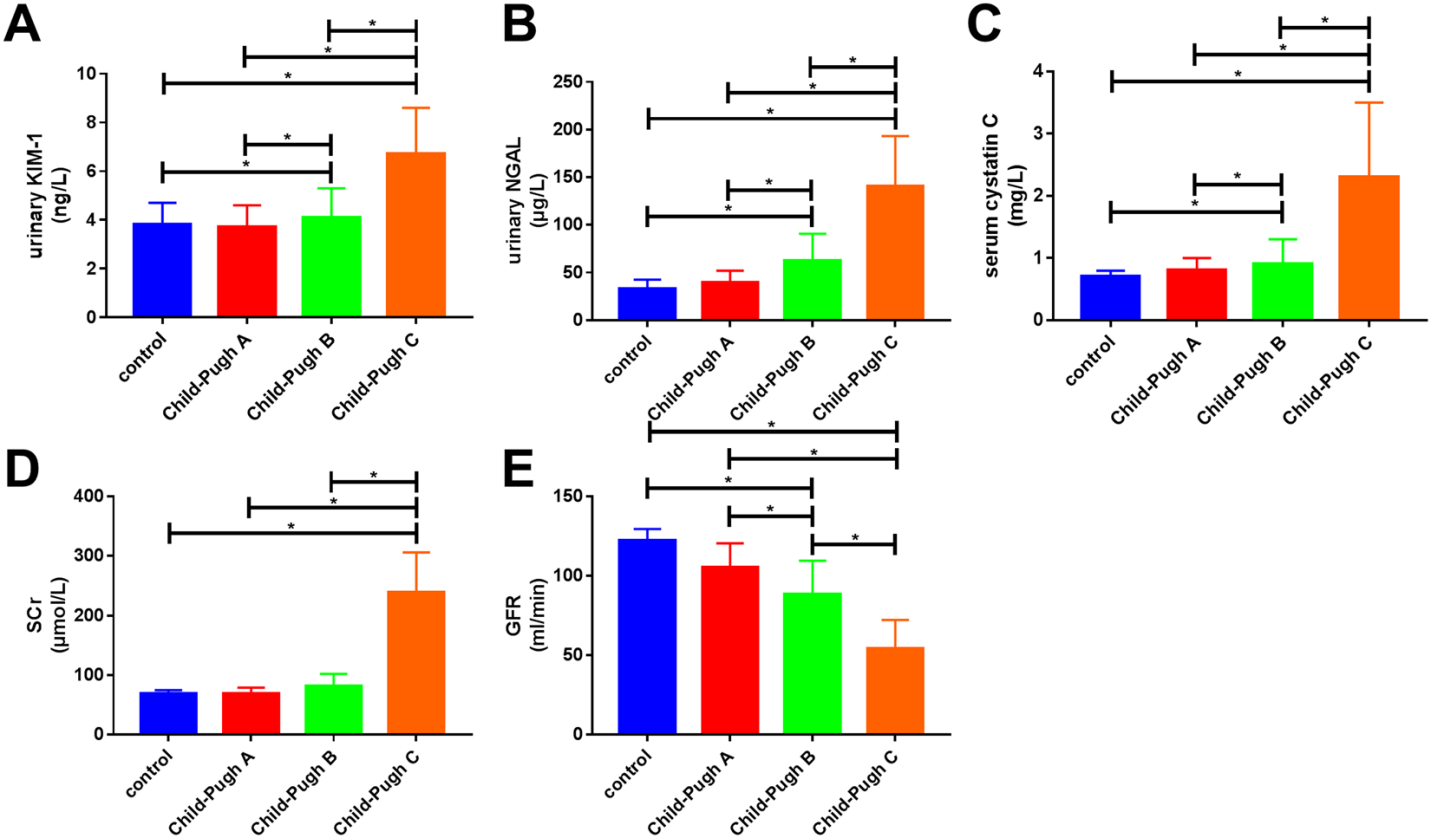
As Child-Pugh class increases in decompensated cirrhosis patients, urinary kidney injury molecule-1 (KIM-1), urinary neutrophil gelatinase-associated lipocalin (NGAL), and serum cystatin C (Cys C) concentrations increase significantly, whereas glomerular filtration rate (GFR) gradually decreases; these differences were statistically significant (*p* < 0.05). However, compared to that in the healthy control group, serum creatinine (SCr) levels in the Child-Pugh Class B patients were not significantly increased (*p* > 0.05), whereas SCr levels in Child-Pugh Class C patients were significantly increased (*p* < 0.05).

Urinary KIM-1, urinary NGAL, serum Cys C, and SCr levels among Child-Pugh Class C patients (9.7 ± 1.9 ng/L, 140.5 ± 52.7 µg/L, 3.3 ± 1.2 mg/L, and 239.1 ± 66.9 µmol/L, respectively) were significantly higher than those in Child-Pugh Class B (4.9 ± 1.2 ng/L, 62.5 ± 28.2 µg/L, 1.7 ± 0.4 mg/L, and 81.1 ± 21.2 µmol/L, respectively) and Class A (3.7 ± 0.9 ng/L, 39.5 ± 12.4 µg/L, 0.8 ± 0.2 mg/L, and 69.3 ± 9.9 µmol/L, respectively) patients, as well as those in the healthy control group (3.1 ± 0.9 ng/L, 33.0 ± 9.7 ng/L, 0.7 ± 0.1 mg/L, and 68.5 ± 6.4 µmol/L, respectively). GFR among Child-Pugh Class C patients (54.2 ± 18.3 mL/min) was significantly lower than that in Child-Pugh Class B (88.5 ± 21.1 mL/min) and Class A (105.4 ± 15.1 mL/min) patients, as well as that in the healthy control group (122.2 ± 7.4 mL/min). All differences were statistically significant (*p* < 0.05). Urinary KIM-1, urinary NGAL, serum Cys C levels among Child-Pugh Class B patients were all significantly higher than those in Class A patients and the healthy control group. GFR among Child-Pugh Class B patients was significantly lower than that in Class A patients and the healthy control group. All differences were statistically significant (*p* < 0.05). However, serum SCr levels among Child-Pugh Class B patients were not significantly different from those of Class A patients or those of the healthy control group (*p* > 0.05). This shows that as Child-Pugh class increases, urinary KIM-1, urinary NGAL, and serum Cys C levels increase significantly. Among Child-Pugh Class B patients, the levels of these three indices were significantly increased compared to those in the healthy control group, at which point the SCr response rate was not sensitive, and SCr levels were significantly increased only in Child-Pugh Class C patients, at which point GFR had already decreased by greater than 50% (Fig. 7).

### Efficacy of urinary KIM-1, urinary NGAL, serum Cys C, and the combined detection factor for the diagnosis of AKI secondary to decompensated cirrhosis

**Figure 8:**
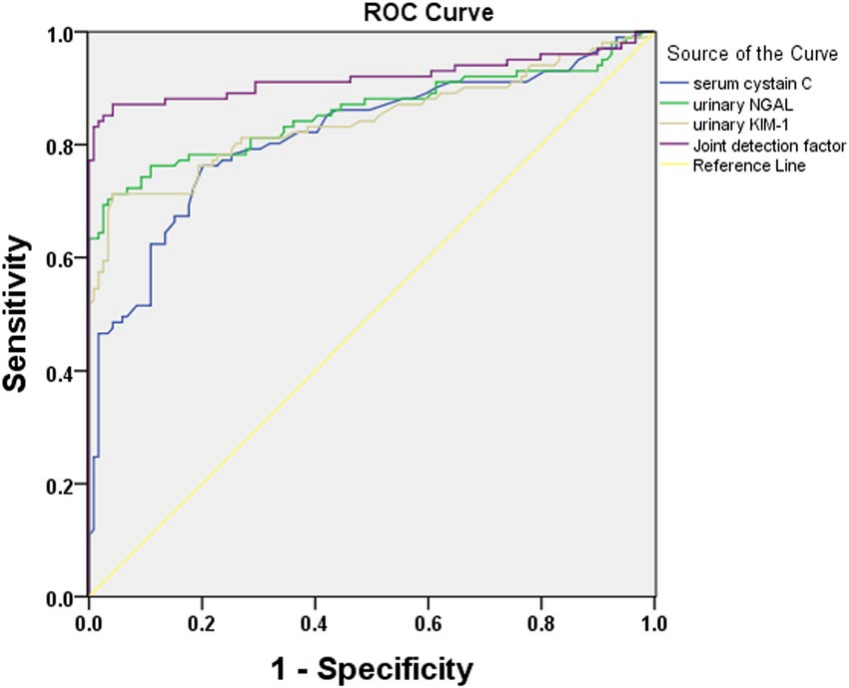
ROC curve of serum cystatin C (Cys C), urinary kidney injury molecule-1 (KIM-1), urinary neutrophil gelatinase-associated lipocalin (NGAL), and the combined detection factor for diagnosis of acute kidney injury (AKI) secondary to decompensated cirrhosis. Urinary KIM-1, urinary NGAL, serum Cys C, and the combined detection factor serving as a screening index for AKI secondary to decompensated cirrhosis had sensitivities of 63.4%, 68.3%, 80.2% and 89.1%, respectively and specificities of 81.6%, 72.1%, 74.9%, and 95.8%, respectively.

An ROC curve was then plotted^13^ using urinary KIM-1, urinary NGAL, serum Cys C, and the combined detection factor as experimental variables and AKI secondary to decompensated cirrhosis (SCr increased by ≥26.5 µmol/L within 48 h) as the state variable. The maximum predictive value obtained served as the clinical threshold of the combined detection factor for diagnosis of AKI secondary to decompensated cirrhosis. The AUC of KIM-1 was 0.843 (95% *CI*: 0.736–0.850, *p* < 0.001), and when urinary KIM-1 concentration was 4.56 ng/L, the sensitivity and specificity were 77.2% and 79.8%, respectively. The AUC of urinary NGAL was 0.857 (95% *CI*: 0.802–0.913, *p* < 0.001), and when urinary NGAL concentration was 53.54 µg/L, the sensitivity and specificity were 78.2% and 80.7%, respectively. The AUC of serum Cys C was 0.816 (95% *CI*: 0.767–0.885, *p* < 0.001), and when serum Cys C concentration was 0.93 mg/L, the sensitivity and specificity were 76.1% and 78.6%, respectively. The AUC of the combined detection factor was 0.919 (95% *CI*: 0.874–0.965, *p* < 0.001), and the sensitivity and specificity were 87.1% and 95.8%, respectively. This shows that urinary KIM-1, urinary NGAL, serum Cys C, and the combined detection factor have predictive value for AKI secondary to decompensated cirrhosis. The combined detection factor had the highest area under the ROC curve, and the sensitivity and specificity were significantly higher than the Youden index. Therefore, the predictive value of the combined detection factor was superior to that of any of the indices alone (Fig. 8).

## Discussion

Acute kidney injury primarily presents as a sharp decrease in GFR, rapid increases in SCr and blood urea nitrogen, and water and sodium retention. It is one of the most common complications of severe liver disease, and particularly end-stage liver disease^14^. Currently, serum creatinine, blood urea nitrogen, urine volume, and other indices are often used clinically to evaluate reductions in kidney function. These indices are not sensitive and are easily affected by non-renal factors. The lack of an effective index for evaluating disease status and prognosis leads to inaccurate clinical evaluation, suboptimal efficacy, and poor prognosis. Moreover, this is an important factor contributing to the high mortality rate of AKI^15,16^. Thus, the search for specific, sensitive early diagnostic markers is essential for treatment, reducing patient mortality, and improving prognosis.

KIM-1 is a transmembrane protein with obvious advantages as a new marker for the early diagnosis of AKI^17^. In normal kidney tissue, it is virtually not expressed^6,7^, but it is expressed at moderate to high levels in the endothelial cells of the proximal convoluted tubules during the early stages of renal ischemic or nephrotoxic injury. It is also correlated with the severity of kidney injury. Moreover, it has strong specificity, especially for ischemic or nephrotoxic AKI, and is rarely expressed in other organs. Furthermore, it has high stability. Further, KIM-1 has protein hydrolytic regions that make it easy to detect in the urine. Studies have shown that in cirrhosis patients, blood KIM-1 levels increase significantly as eGFR levels decrease^18^. The present study also found that urinary KIM-1 concentrations are significantly higher in the group with AKI secondary to decompensated cirrhosis compared to that in the non-AKI and healthy control groups, and that KIM-1 concentrations gradually increase with disease progression, with concentrations highest with Stage 3 AKI. This shows that urinary KIM-1 can serve as an effective marker for monitoring AKI secondary to cirrhosis, and can be an early marker of disease progression.

NGAL is a newly identified member of the lipocalin family^19^. Researchers have found that intraperitoneal injection of high doses of cisplatin, which can result in glomerular necrosis, can rapidly induce NGAL expression in the kidney and its release from the glomeruli. NGAL is expressed in injured glomeruli and can induce epithelial regeneration. It is also generally released into the blood in large amounts within 2 h of injury and excreted via the urine^20^. A study of urinary NGAL levels in 132 cases of decompensated cirrhosis patients by Barreto *et al*.^10^ found that urinary NGAL levels in AKI patients were significantly higher than those in non-AKI patients, NGAL levels with persistent AKI were significantly higher than those with transient AKI, and that it could be used to distinguish hepatorenal syndrome (HRS) from kidney failure due to other causes. A study by Verna *et al*.^11^ found that urinary NGAL concentrations of 110 ng/mL were associated with a sensitivity of 88% and a specificity of 85% for the diagnosis of non-prerenal AKI in decompensated cirrhosis patients. NGAL is an independent predictor of irreversible kidney function injury, and thus might be independent of other commonly used risk factors for predicting patient mortality^21^. The present study also found that urinary NGAL levels were significantly higher in the group with AKI secondary to decompensated cirrhosis than in the non-AKI and healthy control groups, and that urinary NGAL levels increased significantly with AKI progression. The ROC curve showed 53.54 µg/L as the predictive value for the diagnosis of AKI secondary to decompensated cirrhosis, which was associated with high specificity and sensitivity.

Cys C is only eliminated from the body via the kidneys, and kidney microlesions during the early stages can lead to changes in serum Cys C. An increasing number of studies have confirmed that this can serve as a marker for evaluating the status and prognosis of acute kidney injury^22^. In addition, serum Cys C levels are not easily affected by age, sex, race, or body mass; moreover, it is especially worth noting that hyperbilirubinemia does not interfere with serum Cys C monitoring^23^. Cys C > 1.23 mg/L has a sensitivity of 66% and a specificity of 86% for the diagnosis of AKI, and is also a good predictive index for short-term disease-related mortality^24^. Cys C can reflect GFR with more sensitivity than creatinine during a short period of mild to moderate injury, and allows for acute renal failure to be detected earlier, and even with greater diagnostic accuracy, as compared to that with serum creatinine. Studies have shown that upon mild kidney injury, Cys C begins to increase 1–2 d before creatinine increases, and Cys C gradually increases with disease progression^23^. The present study also found that serum Cys C was significantly elevated in the group with AKI secondary to decompensated cirrhosis compared to that in the non-AKI and healthy control groups. In addition, serum Cys C was significantly increased with AKI progression and levels were significantly increased among deceased patients compared to those in living patients. This indicates that serum Cys C can also serve as an effective index for monitoring AKI secondary to cirrhosis.

The results of the present study show that urinary KIM-1, urinary NGAL, and serum Cys C levels gradually increased with increasing disease severity when patients were classified into different stages of AKI. This shows that these three indices are closely related to the severity of AKI secondary to decompensated cirrhosis; specifically, as urinary KIM-1, urinary NGAL, and serum Cys C levels increase, the degree of kidney injury becomes more severe. At the same time, correlation analysis using patients with AKI secondary to decompensated cirrhosis revealed that urinary KIM-1, urinary NGAL, and serum Cys C levels were negatively correlated with GFR, whereas these parameters showed no such correlation in the healthy control group. This confirms that urinary KIM-1, urinary NGAL, and serum Cys C levels can reflect the status of glomerular filtration with high sensitivity.

As Child-Pugh class increased, urinary KIM-1, urinary NGAL, and serum Cys C levels increased significantly. In Child-Pugh Class B patients, the levels of these three indices were all increased by greater than 50% compared to those in the control group, at which point the SCr response rate was not sensitive. SCr levels were significantly increased only in Child-Pugh Class C patients, at which point GFR had already decreased by greater than 50%. This fully explains the high sensitivity of urinary KIM-1, urinary NGAL, and serum Cys C for the diagnosis of AKI secondary to decompensated cirrhosis, and suggests that they can serve as early signs of disease progression.

Based on the roles of urinary KIM-1, urinary NGAL, and serum Cys C in kidney injury, the present study evaluated their value in predicting AKI secondary to decompensated cirrhosis. An ROC curve was plotted using urinary KIM-1, urinary NGAL, serum Cys C, and the combined detection factor as experimental variables and AKI secondary to decompensated cirrhosis (SCr increased by ≥26.5 µmol/L within 48 h) as the state variable. It was found that urinary KIM-1, urinary NGAL, or serum Cys C alone, as well as the combined detection factor, all had predictive value for this condition (all *p* < 0.05). However, the combined detection factor had the highest area under the ROC curve, and the sensitivity and specificity were significantly higher. Therefore, the predictive value of this parameter was superior to any of the indices alone.

Urinary KIM-1, urinary NGAL, and serum Cys C can serve as effective indices for diagnosing AKI secondary to decompensated cirrhosis. These three indices have greater sensitivity than traditional indices for monitoring kidney function, and can be utilized to evaluate the severity and prognosis of AKI. In addition, the combined detection factor has even higher predictive value for AKI secondary to decompensated cirrhosis. In the clinic, these three indices can be easily measured. Moreover, the dynamic observation of urinary KIM-1, urinary NGAL, and serum Cys C levels in decompensated cirrhosis patients allows the physician to understand changes in the disease and assess prognosis, and should be widely implemented. However, because the present study is a single-centre study with a small number of samples, the study is limited to a certain extent, and further confirmation with a large-sample, multi-centre, prospective clinical trial is needed in the future.

## Materials and Methods

### Subjects

Study subjects comprised 150 patients with decompensated cirrhosis hospitalized at the Gastroenterology Department of the Sichuan Provincial People’s Hospital between June 2015 and January 2017. They were divided into an AKI group (n = 68) and a non-AKI group (n = 82) according to dynamic changes in creatinine levels. At the same time, healthy individuals, based on physical examination (n = 70), were selected as a control group. The diagnostic criteria for decompensated cirrhosis were published in the 2012 update of the American Association for the Study of Liver Diseases Practice Guideline for Management of Adult Patients with Ascites Due to Cirrhosis^25^. The inclusion criteria for the AKI group were the Kidney Disease Improving Global Outcomes (KDIGO) criteria^12^, presented as follows: increased SCr ≥26.5 µmol/L within 48 h, SCr increased by ≥ 1.5-fold relative to baseline values within 7 d, or urine volume <0.5 mL/kg/h for 6 continuous hours. The 150 decompensated cirrhosis patients were classified according to Child-Pugh liver function class, with 24 class A patients, 55 class B patients, and 71 class C patients. The exclusion criteria were as follows: (1) history of nephrotoxic medication use; (2) accompanying underlying kidney disease or liver cancer; (3) patients with concomitant conditions that might damage kidney function, including rheumatoid immune disease, diabetes and hypertension, coronary heart disease, or cardiac insufficiency.

### Ethical approval

Informed consent was obtained from all patients, in accordance with the Declaration of Helsinki. This study was approved by the local Ethics Committee of Sichuan Provincial People’s Hospital, and all experiments in this research were performed in accordance with the relevant guidelines and regulations.

### Data collection and biomarker assays

A clinical registration form was designed, and relevant clinical data and laboratory indices were recorded, including age, sex, weight, course of disease, aetiology, relevant complications, urinary KIM-1, urinary NGAL, and serum Cys C, among others. Blood and urine samples from hospitalized decompensated cirrhosis patients were continuously collected. A 5-mL sample of serum was collected from each patient after fasting on the day of hospitalization and immediately centrifuged at 1500 × *g* for 15 min at room temperature. A 0.5-mL aliquot of serum was placed in a sterile centrifuge tube and stored at −80 °C for further analyses. A 20-mL midstream urine sample was collected on the first day after hospitalization and centrifuged at 3000 × *g* for 15 min at room temperature to remove debris. To avoid repeated freeze-thaw cycles, 1.5-mL aliquots of the supernatant were placed in sterile centrifuge tubes and stored at −80 °C until use. During analyses, all samples were thawed only once, and urine precipitates were thoroughly mixed after complete thawing.

Measurement of serum creatinine levels: For this, the sarcosine oxidase assay was used. The Abbott ARCHITECT c16000 automatic biochemical analyzer (USA) and Sichuan Makerbio Co. Ltd. kits (China) were used for measurement.

Measurement of serum Cys C levels: The particle enhanced turbidimetric inhibition immunoassay method was used for this assay. The Abbott ARCHITECT c16000 automatic biochemical analyzer and Biosino Co. Ltd. Kits (China) were used for measurements. At the time of measurement, urine samples stored at −80 °C were thawed at room temperature. The kit, stored at 4 °C, was placed at room temperature. For this assay, Cys C in the sample undergoes an agglutination reaction with anti-human Cys C antibodies in the latex particle suspension, forming an antigen-antibody latex complex and producing turbidity. The level of this turbidity in the presence of a given amount of antibody is directly proportional to the amount of Cys C in the sample. By measuring the absorbance value at a specific wavelength, serum Cys C content was calculated from a calibration curve.

Measurement of urinary KIM-1 levels: For this assay, an ELISA method was used. Wuhan Boster Biological Technology. Ltd. Kits (China) and associated standards were used. At the time of measurement, urine samples that were previously stored at −80 °C were thawed at room temperature. The kit, which was also maintained at 4 °C, was placed at room temperature. A microplate coated with purified antibodies was used and a solid carrier was prepared. Samples or standards, biotinylated KIM-1 antibody, and HRP-labelled avidin were successively added to KIM-1 antibody-coated microplates, and TMB colorimetric substrate was added after thorough washing. For this assay, TMB turns blue with the catalytic action of peroxidase, and is converted to a yellow colour after the addition of acid. The depth of colour in the samples is directly proportional to the amount of KIM-1 in the sample. The absorbance at 450 nm (OD value) was measured using a spectrophotometer, and the concentration in the sample was subsequently calculated.

Measurement of urinary NGAL levels: The ELISA method was used for this. Wuhan Boster Biological Technology. Ltd. Kits (China) and associated standards were also used. At the time of measurement, urine samples stored at −80 °C were thawed at room temperature. A microplate coated with purified NGAL antibodies was used and a solid carrier was prepared. NGAL was successively added to monoclonal antibody-coated microplates, which were bound to HRP-labelled NGAL antibodies, forming antibody-antigen-enzyme-labelled-antibody complexes. TMB colorimetric substrate was added after thorough washing. For this assay, TMB turns blue with the catalytic action of HRP, and is converted to a yellow colour after the addition of acid. The depth of colour in the samples is directly proportional to the amount of NGAL in the sample. The absorbance at 450 nm (OD value) was measured using a spectrophotometer, and the NGAL concentration of the sample was then calculated.

GFR was calculated according to the Cockcroft-Gault formula as follows:

$${\rm{GFR}}=(140-{\rm{age}})\times {\rm{body}}\,{\rm{mass}}/72\times {\rm{SCr}}\times 0.84(0.85\,{\rm{if}}\,{\rm{female}}).$$

### Assessment of renal function

The KDIGO criteria^12^ defines AKI as an increase in SCr by ≥0.3 mg/dL (26.5 μmol/L) within 48 h, an increase in SCr to ≥1.5-fold that of baseline values, which is known or presumed to have occurred within the previous 7 d, or urine volume <0.5 mL/kg/h for 6 h. Stage 1 is defined as an increase in serum creatinine to more than 1.5–1.9-fold greater than baseline values, an increase in SCr ≥0.3 mg/dL (26.5 μmol/L), or urine output <0.5 mL/kg/h for 6–12 h. Stage 2 was defined as an increase in serum creatinine to more than 2.0–2.9-fold greater than baseline values or urine output <0.5 mL/kg/h for ≥12 h. Stage 3 was an increase in serum creatinine to more than 3.0-fold that of baseline values, an increase in SCr to ≥4.0 mg/dL (353.6 μmol/L), the initiation of renal replacement therapy, urine output <0.3 mL/kg/h for ≥24 h, or anuria for ≥12 h.

### Statistical analysis

Epidata 3.1 software was used for data registration. SPSS 19.0 software was used for data analysis. Normally distributed quantitative data are shown as the mean ± standard deviation. Analysis of variance was used to compare differences among multiple groups. The LSD test was used for pairwise comparisons. Pearson correlation analysis was used for the correlation analysis of bivariate normally distributed data. The logistic equation was used to establish a combined detection factor according to the predicted boundary values for each indicator, and then the ROC curve was plotted. The significance level was α = 0.05, and *p* < 0.05 was considered statistically significant.

### Data availability

The datasets generated during and/or analysed during the current study are available from the corresponding author on reasonable request.
